# A phase 1 dose escalation study of BI 831266, an inhibitor of Aurora kinase B, in patients with advanced solid tumors

**DOI:** 10.1007/s10637-014-0201-7

**Published:** 2014-12-23

**Authors:** Christian Dittrich, Michael A. Fridrik, Robert Koenigsberg, Chooi Lee, Rainer-Georg Goeldner, James Hilbert, Richard Greil

**Affiliations:** 1 Ludwig Boltzmann Institute for Applied Cancer Research (LBI-ACR VIEnna) – LB Cluster Translational Oncology and Applied Cancer Research-Institution for Translational Research Vienna (ACR-ITR VIEnna), Third Medical Department, Center for Oncology and Hematology, Kaiser-Franz-Josef-Spital, Vienna, Austria; 2 Center for Hematology and Medical Oncology, General Hospital Linz, Linz, Austria; 3 Boehringer Ingelheim Ltd., Bracknell, Berkshire UK; 4 Boehringer Ingelheim Pharma GmbH & Co. KG, Biberach an der Riß, Germany; 5 Boehringer Ingelheim Pharmaceuticals, Inc, Ridgefield, CT USA; 6 Third Medical Department of Hematology, Medical Oncology, Hemostaseology, Rheumatology and Infectiology, Salzburg Cancer Research Institute, Laboratory for Immunological and Molecular Cancer Research (SCRI-LIMCR) and Center for Clinical Cancer and Immunology Trials (SCRI-CCCIT), Paracelsus Medical University, Salzburg, Austria

**Keywords:** Aurora kinase, Clinical trials, Phase 1, Maximum tolerated dose, Pharmacodynamics, Pharmacokinetics, Solid tumors

## Abstract

*Purpose* BI 831266 is a potent, selective, low-molecular-weight inhibitor of Aurora kinase B. This trial aimed to determine the maximum tolerated dose (MTD) of BI 831266 in patients with advanced solid tumors (NCT00756223; EudraCT 2008-001631-36; 1257.1). *Methods* BI 831266 (4–130 mg) was administered over 24 h on days 1 and 15 of a 4-week schedule. A modified 3 + 3 dose-escalation design was utilized to evaluate the MTD. Safety, pharmacokinetics, pharmacodynamics, objective response rate, progression-free survival (PFS) and exploratory biomarkers were secondary endpoints. *Results* Twenty-five patients received BI 831266. The most frequent tumor type was colorectal cancer (48 %). One patient (130 mg) experienced a dose-limiting toxicity of grade 3 febrile neutropenia. The trial was prematurely terminated (sponsor decision) without further dose-escalation. The most frequent treatment-related adverse events (AEs) were fatigue (20 %), neutropenia, alopecia (16 % each), anemia, dry skin, and nausea (12 % each). Treatment-related grade ≥3 AEs were neutropenia (12 %), anemia (8 %), and febrile neutropenia (4 %); 15 patients experienced serious AEs. High variability in the pharmacokinetic profiles precluded definitive pharmacokinetic conclusions. Exploratory biomarker determination revealed consistency with the mode of action as an Aurora kinase B inhibitor. One patient (4 %; 32 mg) with cervical cancer demonstrated a confirmed partial response (duration 141 days, PFS 414 days). Four patients had stable disease. *Conclusion* The MTD of BI 831266 was not reached because of early trial termination. BI 831266 demonstrated a generally manageable safety profile and signs of antitumor activity in some patients’ solid tumors.

## Introduction

Aurora kinases comprise a family of 3 nuclear serine/threonine kinases (Aurora kinases A, B, and C) that play important roles in maintaining the fidelity of mitosis and genetic stability of cells [1, 2]. Aurora kinase A is involved in mitotic entry, separation of centriole pairs, bipolar spindle assembly, alignment of metaphase, and completion of cytokinesis [1–4]. Aurora kinase B, previously known as AIM-1, regulates chromosomal orientation, chromosome condensation, spindle assembly, and cytokinesis [1, 2, 5]. It also plays a direct role in the phosphorylation of histone H3, which is thought to be principally linked to the initiation of cell division [6, 7] and to chromosome instability and carcinogenesis [8]. While the role of Aurora kinase C is less well characterized, Aurora kinases A and B are known to be involved in tumorigenesis [1, 2]. For example, overexpression of Aurora kinase B induces metastasis after implantation of tumors in nude mice [8]. Furthermore, overexpression of Aurora kinase B has been associated with poor outcome in a variety of tumors including glioblastoma, thyroid, and colon cancers [9–11].

The essential functions of Aurora kinases, along with their aberrant expression across a range of tumor types, has resulted in numerous small molecule inhibitors currently being investigated as potential therapies for solid and hematologic tumors [1, 2]. One of these agents is BI 831266, a potent and selective low-molecular-weight inhibitor of Aurora kinase B. Preclinical studies show that BI 831266 inhibits the proliferation of human non-small cell lung cancer (NSCLC), pancreatic cancer, and prostate cancer cell lines. Furthermore, in murine xenograft tumor models (HCT 116 colon carcinoma, BxPC3 pancreatic adenocarcinoma, and NCI-H460 NSCLC), a 24-h continuous infusion of BI 831266 resulted in tumor regression and growth inhibition (data on file, Boehringer Ingelheim).

Here, we report the results from a multicentric, phase 1 trial of single-agent BI 831266. The purpose of this study was to determine the maximum tolerated dose (MTD) of BI 831266 administered to patients with a range of advanced solid tumors, and to assess the safety and tolerability of the compound. The pharmacokinetic (PK) and pharmacodynamic profiles and the clinical antitumor activity of BI 831266 were also investigated. In an exploratory approach, the role of inhibiting the phosphorylation of histone H3 in the skin and caspase cleaved fragment of cytokeratin-18 (CK-18) in plasma were investigated as potential biomarkers. Furthermore, a potential dependence of exposure to BI 831266 on endogenous alpha-1-acid glycoprotein (AGP) concentration was investigated.

## Methods

### Patient selection

Patients ≥18 years of age with advanced, non-resectable and/or metastatic solid malignant tumors, and a life expectancy of ≥3 months were eligible for inclusion in this study. Patients had failed conventional treatment, were not amenable to established treatment options, or there was no therapy of proven efficacy available. Other eligibility criteria included: Eastern Cooperative Oncology Group performance status ≤2; recovery from toxicities from previous treatments to Common Terminology Criteria for Adverse Events (CTCAE) grade ≤1; adequate bone marrow, liver, and renal function (absolute neutrophil count ≥1500/mm^3^, platelet count ≥100,000/mm^3^, bilirubin ≤1.5 mg/dL [≤26 μmol/L, SI unit equivalent], aspartate aminotransferase and/or alanine aminotransferase ≤2.5 times the upper limit of normal; if related to liver metastases, ≤5 times the upper limit of normal, serum creatinine ≤1.5 mg/dL [≤132 μmol/L, SI unit equivalent]); no chemotherapy, radiotherapy, immunotherapy, hormone therapy, or investigational therapy within 2 weeks prior to the start of treatment with the trial drug; no symptomatic brain metastases, leptomeningeal disease, or second malignancy requiring therapy; and no serious illness or concomitant disease that could potentially compromise patient safety (including clinically significant cardiovascular disease and/or a left ventricular ejection fraction <50 %). Also, it was required that patients had a secure central venous access.

During the study, treatment with corticosteroids was permitted. Use of growth factors, such as granulocyte colony stimulating factor, was also allowed for treatment of prolonged hematotoxicity at the investigator’s discretion, but not in cycle 1 of treatment unless medically necessary. Ongoing treatment with bisphosphonates or gonadotropin-releasing hormone analogs for prostate cancer could be continued in the trial, and concomitant therapies to provide adequate care were given as deemed clinically necessary.

All patients were required to provide written informed consent consistent with International Conference on Harmonization–Good Clinical Practice guidelines and local legislation.

### Study design and endpoints

This was an open-label, phase 1, dose-escalation trial of BI 831266 in patients with advanced solid tumors conducted at 3 sites in Austria (NCT00756223; EudraCT 2008-001631-36; 1257.1). The trial was conducted in accordance with the principles laid down by the Declaration of Helsinki and approved by the Independent Ethics Committees and/or Institutional Review Boards of the participating centers.

A modified 3 + 3 dose-escalation design was used to evaluate the MTD of BI 831266, which was administered by intravenous infusion over 24 h (via central venous access) on days 1 and 15 of a 4-week schedule. Initially, 2 treatment schedules had been planned: a 4-week schedule and a 3-week schedule (day 1, every 3 weeks). After the MTD for the 4-week schedule had been established, recruitment to determine the MTD of BI 831266 on a 3-week schedule was to be initiated, with dose tiers starting at the MTD determined in the 4-week schedule. However, for reasons stated in the Results section and presented in detail in the Discussion, the 3-week schedule was not started.

Cohorts of 3–6 patients were enrolled sequentially into escalating dose tiers of BI 831266. The MTD was defined as the highest dose of BI 831266 at which no more than 1 of 6 patients experienced a dose-limiting toxicity (DLT) during the first cycle of treatment. Upon determination of the MTD, entry of additional patients at this dose level, in the form of an expansion cohort, was planned to obtain additional safety data.

The safe starting dose was calculated based on the FDA guidance for starting dose selection for a cytotoxic agent in cancer patients. Based on the data from the 3-cycle toxicity study in rats, the dose severely toxic to 10 % (STD10) of the animals was 4.5 mg/kg which is equivalent to 27 mg/m^2^ body surface area. One tenth of this rodent dose was not severely toxic to dogs in the 3-cycle toxicity study in dogs. One tenth of the STD10 in rats, 2.7 mg/m^2^, is equivalent to 4.3 mg per patient (based on a body surface area of 1.6 m^2^ per patient). Therefore, 4 mg was determined as the safe starting dose in humans. Dose escalation occurred in steps of 100 % until the observation of the first drug-related grade ≥2 adverse event (AE) in cycle 1. Thereafter, doses of BI 831266 were intended to be escalated in decreasing steps of 100 to 20 % (or, prior to the protocol amendment dated July 20, 2009: ≤50 % until the first DLT, and ≤35 % thereafter). Patients were treated for as long as clinical benefit was derived. Treatment was terminated if a DLT occurred which did not resolve to a degree that allowed treatment continuation; if a second DLT occurred; if there was an intolerable AE; if consent was withdrawn; or if a treatment cycle was delayed for >2 weeks.

One protocol amendment was made during the study: the dose escalations were changed from ‘100 % until the first CTCAE grade ≥2, then ≤50 % until the first DLT, then ≤35 % thereafter’, to ‘100 % until the first DLT followed by 100 to 20 % thereafter, depending on the overall safety information, including previous dose cohorts and cycles ≥2’. This amendment was performed to make the dose escalation more flexible and faster, and to reduce the number of patients unnecessarily treated at low (and possibly sub-therapeutic) doses of BI 831266 in the early part of the study. This was supported by the safety data from the on-going phase 1 study of the sister front-runner compound BI 811283 [12], which was already recruiting patients at much higher doses (105 mg in Schedule A) using the same treatment schedules as in this study and a similar starting dose, with no significant safety issues. The amendment also changed the criterion for starting the 3-week treatment schedule from ‘the occurrence of the first relevant drug-related AE’ to ‘the establishment of the MTD in the 4-week schedule’. The reason for this amendment was to reduce the overall number of patients treated in the study, again based on the data from the ongoing phase 1 study of the front-runner compound BI 811283, which was already treating more patients than anticipated due to the unexpectedly high number of dose steps in that study. Thus, it was decided not to recruit patients into the 3-week schedule of BI 831266 until the MTD for the 4-week schedule had been established.

The primary endpoint of the trial was the MTD of BI 831266, administered as a 24-h continuous infusion in a 4-week schedule. Secondary endpoints included incidence and intensity of AEs; incidence of DLTs; the PK profile of BI 831266; progression-free survival (PFS); objective response rate; and duration of response. Pharmacodynamic endpoints included change from baseline in the percentage of epidermal cells expressing phosphorylated histone H3 (pHH3) and plasma concentrations of caspase-cleaved fragment of CK-18 and AGP.

### Definition of DLT

A DLT was defined as one of the following events occurring during the first treatment cycle: a drug-related non-hematologic toxicity grade ≥3 (except untreated nausea, vomiting, or diarrhea); drug-related neutropenia lasting for ≥7 days or febrile neutropenia grade 4; or drug-related thrombocytopenia grade 4 or thrombocytopenia grade ≥3 with complications.

### Assessments

Safety was assessed as incidence and intensity of AEs, using Medical Dictionary for Regulatory Activities criteria and graded according to CTCAE version 3.0. Changes in safety tests, including vital signs (blood pressure and pulse rate were evaluated every 2 h during infusion of BI 831266), echocardiograms, electrocardiograms, and laboratory tests, were also assessed.

Objective tumor response was measured using CT and/or MRI scans performed at baseline and at the end of every other treatment course, and evaluated according to Response Evaluation Criteria in Solid Tumors (RECIST) version 1.0 [13]. The duration of overall response was measured from the time at which the measurement criteria were met for complete response or partial response (PR; whichever was recorded first) until the first date that recurrent or progressive disease (PD) was objectively documented. PFS was defined as the duration of time from the start of treatment to the time of progression or death.

Plasma samples for PK analysis were collected for the first and second infusions of BI 831266 at the following times: 5 min before the start of the 24-h infusion and 01:00, 02:00, 04:00, 12:00, 20:00, 23:59 (just before the end of the infusion), 24:15, 24:30, 25:00, 26:00, 28:00, 32:00, 48:00, 72:00, and 168:00 h after the start of the infusion. Further samples were obtained in subsequent treatment cycles up to cycle 6, just prior to the start and the end of the infusion of BI 831266. Urine samples were also obtained after the first and second infusions of BI 831266 at 0–4 h, 4–12 h, 12–24 h, 24–48 h, and 48–72 h after the start of infusion. Concentrations of BI 831266 in plasma and urine were determined using a validated high-performance liquid chromatography–tandem mass spectrometry assay (data on file, Boehringer Ingelheim). Quantification was performed using a weighted (1/*x* ^2^) linear least-squares regression analysis generated from calibration standards. For plasma samples, the lower limit of quantification for BI 831266 was 0.2 nM and the assay had a linear range up to 200 nM. For urine samples, the lower limit of quantification for BI 831266 was 2.00 nM and the assay had a linear range up to 2000 nM. The mean relative error of the quality control samples was 0.6–2.8 % and the mean coefficient of variation range was 5.2–5.9 %.

Histone H3 is a well characterized substrate of Aurora B, and measurement of its phosphorylation on serine 10 may be used as a pharmacodynamic biomarker of Aurora B inhibition [14]. In a previous study (data on file, Boehringer Ingelheim) [15] a method had been described that allowed parallel determination of the pHH3 content in skin biopsies using Western Blot and immunohistochemistry analyses. This method was included in this study to analyze patient’s skin biopsies, taken before and after treatment, with the expectation that there should be a reduction in pHH3 post-treatment. Epidermal pHH3 expression was determined in skin biopsies obtained during screening and on day 16 of the first 4-week treatment cycle, as soon as possible (within 6 h) after the end of the second infusion of BI 831266 at TARGOS Molecular Pharmacology GmBH (Kassel, Germany). Forearm punch biopsies of 3–4 mm were fixed in formaldehyde prior to being paraffin-embedded. Sections were stained for hematoxylin and eosin to identify areas of well preserved, healthy epidermis. Automated immunohistochemical staining for pHH3 was performed (Benchmark XT, Ventana/Roche Tissue Diagnostics, Basel, Switzerland), utilizing a standard Universal DAB detection procedure. Sections were counterstained in hematoxylin II solution. The number of cells with nuclear staining of pHH3 (intensity 3+) per mm of stratum germinativum was counted, and the percentage decrease from baseline in pHH3+ cells/mm of epidermis with BI 831266 was calculated.

Caspase-cleaved fragment of CK-18 is a marker for tumor cell apoptosis. A specific antibody (M30) recognizes a neo-epitope of CK-18 generated during apoptosis. This marker for apoptosis was investigated in an exploratory manner in this study, with the expectation that it should rise following treatment if the treatment causes tumor cell apoptosis. Caspase-cleaved fragments of CK-18 were quantified in plasma samples obtained predose and at 48, 72, and 168 h after the first 2 infusions of BI 831266. Samples were also collected predose and at 168 h post-dose up to cycle 6. Plasma concentrations of caspase-cleaved CK-18 were determined using a validated enzyme-linked immunosorbent assay (ELISA) at Nuvisan GmbH (Neu-Ulm, Germany) and a monoclonal antibody M30-Apoptosense® ELISA kit (Peviva AB, Bromma, Sweden). The assay method is a sandwich type immunoassay that uses the monoclonal ‘M5’ capture antibody directed against CK-18 and a horseradish peroxidase conjugated monoclonal ‘M30’ antibody directed against the CK-18 asparate 396 neo-epitope.

BI 831266 showed a moderate binding to plasma proteins in humans, dogs and rats that was independent of the BI 831266 concentration. BI 831266 is bound to human serum albumin as well as to AGP. Binding to AGP was highly dependent on the AGP concentration. Higher AGP led to higher total exposures of BI 831266 in plasma. To explore the relevance of this finding, AGP plasma levels were investigated in this study. AGP was quantified in plasma samples obtained during the first 2 treatment cycles at the same time points as those used for the PK analysis. Further samples were obtained up to cycle 6, predose, and at 168 h post-dose. AGP concentrations were determined using a validated immunoturbidimetric assay.

### Statistical analyses

All statistical analyses were descriptive and exploratory in nature. A single population was defined for the efficacy and safety analyses, which included all patients who received at least 1 dose of BI 831266. All treated patients were also included in the PK analysis, but patients with PK data that did not appear plausible, or those with insufficient data, were excluded from PK analyses.

## Results

### Patient demographics and disposition

Of the 29 patients who were enrolled in this trial, 25 were subsequently entered and treated with study medication between November 2008 and October 2010. The most frequent tumor type was colorectal cancer (CRC; *n* = 12, 48 %). All patients had undergone prior surgery and all patients except for 1 had received prior chemotherapy for metastatic disease. Most patients were heavily pretreated, with 18 patients (72 %) having received ≥3 lines of prior chemotherapy (Table 1).

**Table 1:** Patient demographics and disease characteristics at baseline

	Total patients
	(*N* = 25)
Median age, years (range)	66 (39–79)
Gender, *n* (%)	
Male	16 (64.0)
Female	9 (36.0)
Baseline ECOG PS, *n* (%)	
0	15 (60.0)
1	9 (36.0)
2	1 (4.0)
Type of cancer, *n* (%)	
Colorectal	12 (48.0)
Pancreas	4 (16.0)
Liver and biliary tree	2 (8.0)
Sarcoma of soft tissue or bone	2 (8.0)
Bladder	1 (4.0)
Cervix, vagina, vulva	1 (4.0)
Kidney and ureter	1 (4.0)
Prostate	1 (4.0)
Unknown	1 (4.0)
Prior anticancer therapy, *n* (%)	
Chemotherapy^a^	24 (96.0)
≥ 3 chemotherapies	18 (72.0)
Surgery	25 (100.0)
Radiotherapy	7 (28.0)
Hormone therapy^b^	3 (12.0)
Immunotherapy^b^	5 (20.0)
Other^b^	3 (12.0)

*ECOG PS* Eastern Cooperative Group performance status

^a^Patients had received up to 7 lines of prior chemotherapy for metastatic disease

^b^Data missing for *n* = 2 patients

### Treatment exposure

Patient cohorts were treated with escalating doses of BI 831266: 4 mg (*n* = 4), 8 mg (*n* = 3), 16 mg (*n* = 3), 32 mg (*n* = 3), 64 mg (*n* = 5), and 130 mg (*n* = 7). One patient who received both the day 1 and day 15 infusions died while still on treatment prior to the end of cycle 1 due to PD with multi-organ failure, which was not considered to be drug-related; this patient was therefore not evaluable for DLT. The median number of cycles completed was 2 (minimum: 1 cycle; maximum: 14 cycles; Table 2). Twenty-four patients (96.0 %) discontinued treatment because of PD.

**Table 2:** Exposure to BI 831266

	BI 831266 dose						
	4 mg	8 mg	16 mg	32 mg	64 mg	130 mg	Total
Patients treated, *n* (%)	4 (100)	3 (100)	3 (100)	3 (100)	5 (100)	7 (100)	25 (100)
Number of cycles completed^a^, *n* (%)							
1	0	2 (66.7)	0	0	1 (20)	2^b^ (28.6)	5 (20)
2	3 (75)	0	3 (100)	2 (66.7)	2 (40)	4 (57.1)	14 (56)
3	0	1 (33.3)	0	0	0	0	1 (4)
4	0	0	0	0	1 (20)	1 (14.3)	2 (8)
6	1 (25)	0	0	0	0	0	1 (4)
10	0	0	0	0	1 (20)	0	1 (4)
14	0	0	0	1 (33.3)	0	0	1 (4)
Total exposure time (days)							
Mean	90.5	56.0	65.0	181.0	104.4	57.0	87.6
Minimum, maximum	47, 185	37, 93	65, 65	64, 414	37, 289	23, 109	23, 414

^a^A cycle was defined as completed if the patient received both infusions of BI 831266

^b^One patient received both the day 1 and day 15 infusions of BI 831266 but died before the end of cycle 1

### DLTs, safety, and tolerability

Only 1 patient (treated with 130 mg BI 831266) experienced a DLT: treatment-related grade 3 febrile neutropenia associated with grade 3 reduction in white cell count in cycle 1 starting on day 15 (second infusion). This patient had received 7 prior lines of treatment. The second BI 831266 infusion was completed as planned and the patient’s fever resolved on day 17 following treatment with standard therapy, including filgrastim. Blood counts recovered on day 18 and the patient completed a second cycle of treatment at a reduced dose of 100 mg according to the protocol. There were no other DLTs in the 130 mg cohort. The trial was terminated prematurely by the sponsor without further dose escalation at this stage; therefore, the MTD of BI 831266 in the 4-week treatment schedule could not be determined. The rationale for terminating the trial is described in the Discussion.

Among 25 patients treated, 17 (68 %) patients experienced treatment-related AEs. Across all treatment cycles the most frequent treatment-related AEs were fatigue (20 %), neutropenia and alopecia (16 % each), and anemia, dry skin, and nausea (12 % each; Table 3). Most treatment-related AEs were mild (grade 1/2), with higher grade treatment-related AEs (grade 3/4) observed only at 130 mg, the highest dose administered. All treatment-related grade 3/4 AEs were hematologic and included neutropenia (grade 3: *n* = 2; grade 4: *n* = 1), anemia (grade 3: *n* = 2), and febrile neutropenia (grade 3: *n* = 1; Table 3). The treatment-related grade 4 neutropenia lasted less than 7 days and was not considered a DLT. More hematologic AEs (neutropenia, anemia, thrombocytopenia, febrile neutropenia) and alopecia were observed with 130 mg BI 831266 than with the lower doses. All 7 patients treated with 130 mg BI 831266 experienced at least 1 drug-related hematologic AE, whereas no patients treated at lower doses (4–64 mg) experienced drug-related AEs. In addition, drug-related alopecia was reported more frequently in the 130 mg dose group than in the lower dose groups (Table 3).

**Table 3:** Treatment-related AEs occurring in any patient (total number of patients treated *N* = 25)

Treatment-related AEs in all patients														
BI 831266 dose cohort	4 mg		8 mg		16 mg		32 mg		64 mg		130 mg		Total	
	(*n* = 4)		(*n* = 3)		(*n* = 3)		(*n* = 3)		(*n* = 5)		(*n* = 7)		(*N* = 25)	
*n* (%)	All grades	Grade 3/4	All grades	Grade 3/4	All grades	Grade 3/4	All grades	Grade 3/4	All grades	Grade 3/4	All grades	Grade 3/4	All grades	Grade 3/4
Total number of patients with treatment-related AE	1	0	2	0	3	0	2	0	2	0	7	5	17 (68)	5 (20)
Fatigue	–	–	1	–	–	–	1	–	1	–	2	–	5 (20)	–
Neutropenia	–	–	–	–	–	–	–	–	–	–	4	3	4 (16)	3 (12)
Alopecia	–	–	–	–	–	–	1	–	–	–	3	–	4 (16)	-
Anemia	–	–	–	–	–	–	–	–	–	–	3	2	3 (12)	2 (8)
Dry skin	1	–	–	–	–	–	–	–	1	–	1	–	3 (12)	–
Nausea	–	–	1	–	1	–	–	–	–	–	1	–	3 (12)	–
Thrombocytopenia	–	–	–	–	–	–	–	–	–	–	2	–	2 (8)	–
Abdominal pain	–	–	1	–	–	–	–	–	–	–	1	–	2 (8)	–
Myalgia	–	–	1	–	–	–	–	–	–	–	1	–	2 (8)	–
Febrile neutropenia	–	–	–	–	–	–	–	–	–	–	1	1^a^	1 (4)	1 (4)
Stomatitis	–	–	–	–	–	–	–	–	–	–	1	–	1 (4)	–
Pyrexia	–	–	–	–	–	–	–	–	–	–	1^b^	–	1 (4)	–
Arthralgia	–	–	–	–	–	–	–	–	–	–	1	–	1 (4)	–
Dysgeusia	–	–	–	–	–	–	–	–	–	–	1	–	1 (4)	–
Paresthesia	–	–	–	–	–	–	1	–	–	–	–	–	1 (4)	–
Change of bowel habit	–	–	–	–	1	–	–	–	–	–	–	–	1 (4)	–
Gynecomastia	–	–		–	1	–	–	–	–	–	–	–	1 (4)	–
Abdominal discomfort	–	–	1	–	–	–	–	–	–	–	–	–	1 (4)	–
Abdominal pain lower	–	–	1	–	–	–	–	–	–	–	–	–	1 (4)	–
Scratch	–	–	1	–	–	–	–	–	–	–	–	–	1 (4)	–

*AE* adverse event; *DLT* dose-limiting toxicity

^a^DLT

^b^Treatment-related serious AE

Serious AEs were reported in 15 patients (Table 4); 12 of these patients required hospitalization, with prolonged hospitalization required for 6 patients, and 5 patients had serious AEs resulting in death. One patient died as a result of renal and hepatic failure (4 mg cohort), 1 died because of multi-organ failure (32 mg cohort), and 3 died because of malignant neoplasm progression (64 mg: *n* = 2; 130 mg: *n* = 1). None of these fatal AEs were considered to be drug-related.

**Table 4:** All-causality serious AEs occurring in any patient

BI 831266 dose (mg)	Serious AE per patient
4	Dyspnea, hepatic and renal failure^a^
4	Subileus, pyrexia
8	Ascites
8	Fatigue
32	Gangrene, deep vein thrombosis, chest pain, multi-organ failure^a^
32	Fatigue
64	Catheter site infection, dyspnea, malignant neoplasm progression^a^
64	Infection
64	Hepatic pain, pyrexia, malignant neoplasm progression^a^
64	Diarrhea
64	Gastric hemorrhage
130	Dyspnea, general physical health deterioration, malignant neoplasm progression^a^
130	Abdominal pain, chest pain
130	Jaundice
130	Pyrexia^b^

*AE* adverse event

^a^Serious AE resulting in death

^b^Considered by the investigators to be related to trial medication

Treatment discontinuation occurred in only 1 patient (who was treated at 130 mg). This patient suffered a grade 3 general physical health deterioration, which was not drug-related, and subsequent disease progression.

CTCAE grade 3 white blood cell count was reported in 3 patients in the 130 mg dose group. There was no trend towards reduction of leucocytes or neutrophils over the treatment period.

### Pharmacokinetics

**Fig. 1:**
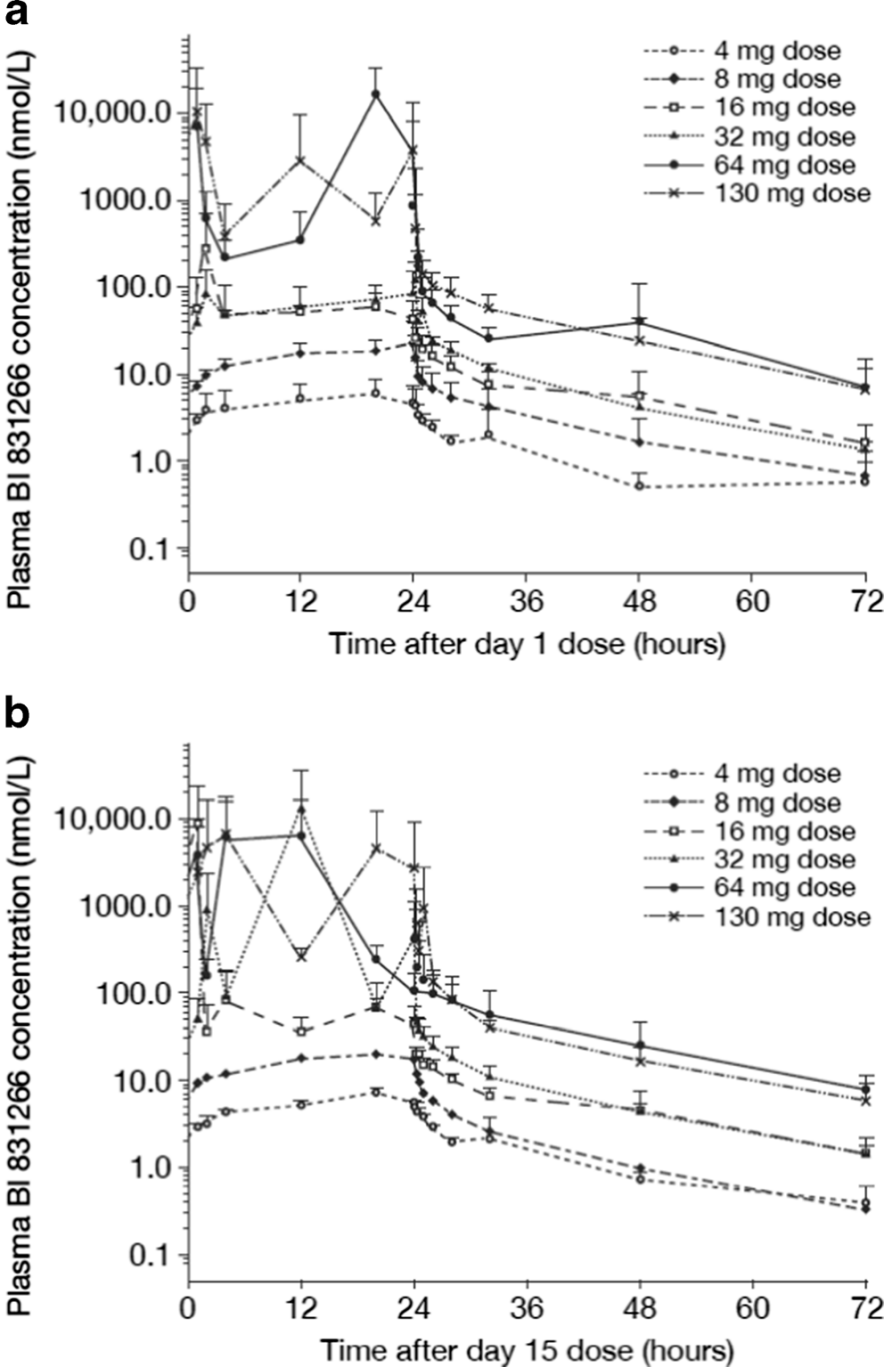
Mean plasma concentration-time profiles of BI 831266 on **a** day 1 and **b** day 15 of cycle 1 after 24-h infusion of 4, 8, 16, 32, 64 and 130 mg BI 831266. Footnote: Concentration-time data could only be evaluated for 2 out of 3 patients in the 8 mg cohort on day 15, therefore standard deviation values could not be calculated

The PK characteristics of BI 831266 during cycle 1 are summarized in Table 5. Plasma concentrations of BI 831266 generally increased during the 24-h infusion of each dose on day 1 or day 15; there was a slight increase of the first 4 dose levels until 4–20 h after the start of the infusion. Time from dosing to maximum measured concentration (t_max_) was reached at ~20 h after the start of the infusion. After the infusion was stopped, plasma concentrations of BI 831266 declined in a biphasic fashion, with an initial rapid decline followed by a much slower elimination phase (BI 831266 plasma concentration-time profiles are shown in Fig. 1). The geometric mean terminal elimination half-life ranged from 15 to 39 h after the first dose and from 14 to 29 h after the second dose. As a consequence of high variability in the BI 831266 exposure profiles, dose proportionality could not be determined. Ten PK profiles did not appear plausible, since plasma concentrations rose and declined extremely rapidly (data not shown). In all treatment groups, the amount of BI 831266 excreted in urine accounted for <15 % of the dose, indicating urinary excretion is a minor pathway of elimination, and nearly all of the BI 831266 that would have been excreted in urine was eliminated within 72 h.

**Table 5:** Non-compartmental PK parameters (geometric means ± geometric coefficients of variation, unless otherwise stated) for the first and second infusions of BI 831266

	4 mg (*n* = 4)	8 mg (*n* = 3)	16 mg (*n* = 3)	32 mg (*n* = 3)	64 mg (*n* = 5)	130 mg (*n* = 7)
Cycle 1, day 1						
C_max_ (nmol/L)	5.84 ± 53	22.0 ± 72	88.2 ± 582	126 ± 43	3310 ± 4080	923 ± 1610
C_max, norm_ (nmol/L)	1.46 ± 53	2.75 ± 72	5.51 ± 582	3.92 ± 43	51.7 ± 4080	7.10 ± 1610
t_max_ (hours)^a^	20.4 (20–24)	23.6 (20–24)	20.0 (2.0–21)	24.3 (20–25)	20.0 (20–48)	20.1 (1.0–22)
CL (mL/min)	858 ± 66	531 ± 42	410 ± 138	544 ± 15	50.4 ± 660	311 ± 307
AUC_0–24_ (nmol∙hours/L)	98.7 ± 64	359 ± 35	928 ± 155	1430 ± 18	29,300 ± 1540	10,400 ± 371
AUC_0–∞_ (nmol∙hours/L)	147 ± 66	475 ± 42	1230 ± 138	1860 ± 15	40,100 ± 660	13,200 ± 307
AUC_0–∞, norm_ (nmol∙hours/L)	36.8 ± 66	59.4 ± 42	77.0 ± 138	58.0 ± 159	626 ± 660	102 ± 307
t_1/2_ (hours)	19.6 ± 71	14.5 ± 18	21.0 ± 59	16.9 ± 64	39.0 ± 30	24.1 ± 42
Cycle 1, day 15						
C_max_ (nmol/L)	6.89 ± 16	–	280 ± 220,000	600 ± 71,200	1260 ± 2600	1080 ± 1370
C_max, norm_ (nmol/L)	1.72 ± 16	–	17.5 ± 220,000	18.8 ± 71,200	19.6 ± 2600	8.31 ± 1370
t_max_ (hours)^a^	20.1 (20–21)	–	20.0 (1.0–21)	20.0 (12–21)	19.1 (12–24)	20.2 (20–24)
CL (mL/min)	649 ± 22	–	219 ± 582	118 ± 4810	109 ± 949	386 ± 245
AUC_0–24_ (nmol∙hours/L)	119 ± 6.5	–	1760 ± 898	7200 ± 7270	13,200 ± 1810	13,200 ± 540
AUC_0–∞_ (nmol∙hours/L)	194 ± 22	–	2300 ± 582	8540 ± 4810	18,500 ± 949	10,600 ± 245
AUC_0–∞, norm_ (nmol∙hours/L)	48.6 ± 22	–	144 ± 582	267 ± 4810	289 ± 949	81.9 ± 245
t_1/2_ (hours)	23.8 ± 108	–	24.2 ± 40	13.8 ± 6.3	26.1 ± 25	29.0 ± 65.3

^a^Median (minimum, maximum)

–Insufficient data to calculate descriptive statistics

*AUC*_*0*–*24*_ area under the plasma concentration–time curve over the time interval from 0 to 24 h after the start of infusion; *AUC*_*0*–∞_ area under the plasma concentration–time curve over the time interval from 0 extrapolated to infinity; *CL* total clearance of analyte in plasma after intravenous administration; *C*_*max*_ maximum measured concentration; _*norm*_ dose normalized; *PK* pharmacokinetic; *t*_*1*/*2*_ terminal half-life; *t*_*max*_ time from dosing to maximum measured concentration

### Pharmacodynamics

**Fig. 2:**
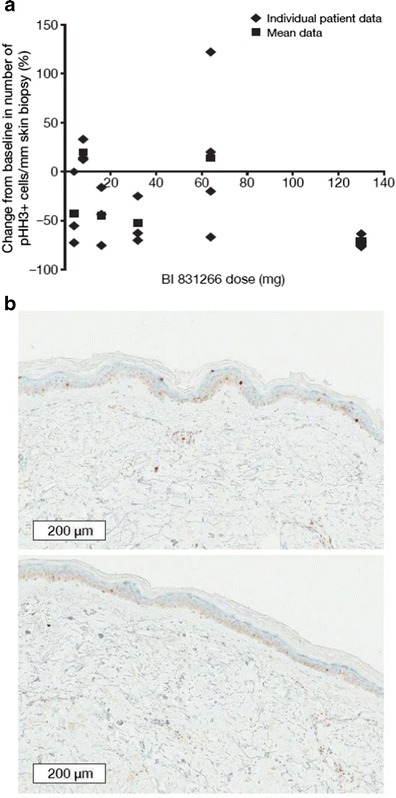
**a** Individual and mean decrease in the number of pHH3+ cells at screening and after infusion of BI 831266 by dose. **b** Immunohistochemical staining for pHH3 at baseline (*top*) and post-treatment (*bottom*) on cycle 1 day 16 in a patient with cervical cancer who experienced a confirmed PR. *pHH3* phosphorylated histone H3, *PR* partial response

Twenty-two (88 %) patients were evaluable for analysis of the change in pHH3 expression in skin biopsies (4 mg: *n* = 3/4; 8 mg, 16 mg, 32 mg: *n* = 3/3 each; 64 mg: *n* = 4/5; 130 mg: *n* = 6/7) and analysis of the change in plasma concentrations of caspase-cleaved CK-18 and AGP (4 mg: *n* = 4/4; 8 mg: *n* = 2/3; 16 mg, 32 mg, 64 mg: *n* = 3/3 each; 130 mg: *n* = 7/7). Mean and individual percentage changes in epidermal cells/mm positive for pHH3 from baseline to post-treatment with BI 831266 are shown in Fig. 2a. There was a general trend towards a decrease in pHH3 with increasing doses of BI 831266. However, high interpatient variability was observed at all doses, except the 130 mg dose. At this dose, there was a mean reduction of 71.6 % (standard deviation, 4.5) in the number of pHH3+ cells/mm, and this reduction was consistent and robust. Immunohistochemical staining images for pHH3 at baseline and post-treatment at cycle 1 day 16 are shown in Fig. 2b for a patient with cervical cancer treated with 32 mg BI 831266 and who had a PR (described below). Each individual patient’s percentage decrease was plotted against the patient’s PK area under the concentration-time curve (AUC) for the dose at which the skin biopsy sample was taken. This did not show an apparent relationship between percentage decrease and drug exposure.

**Fig. 3:**
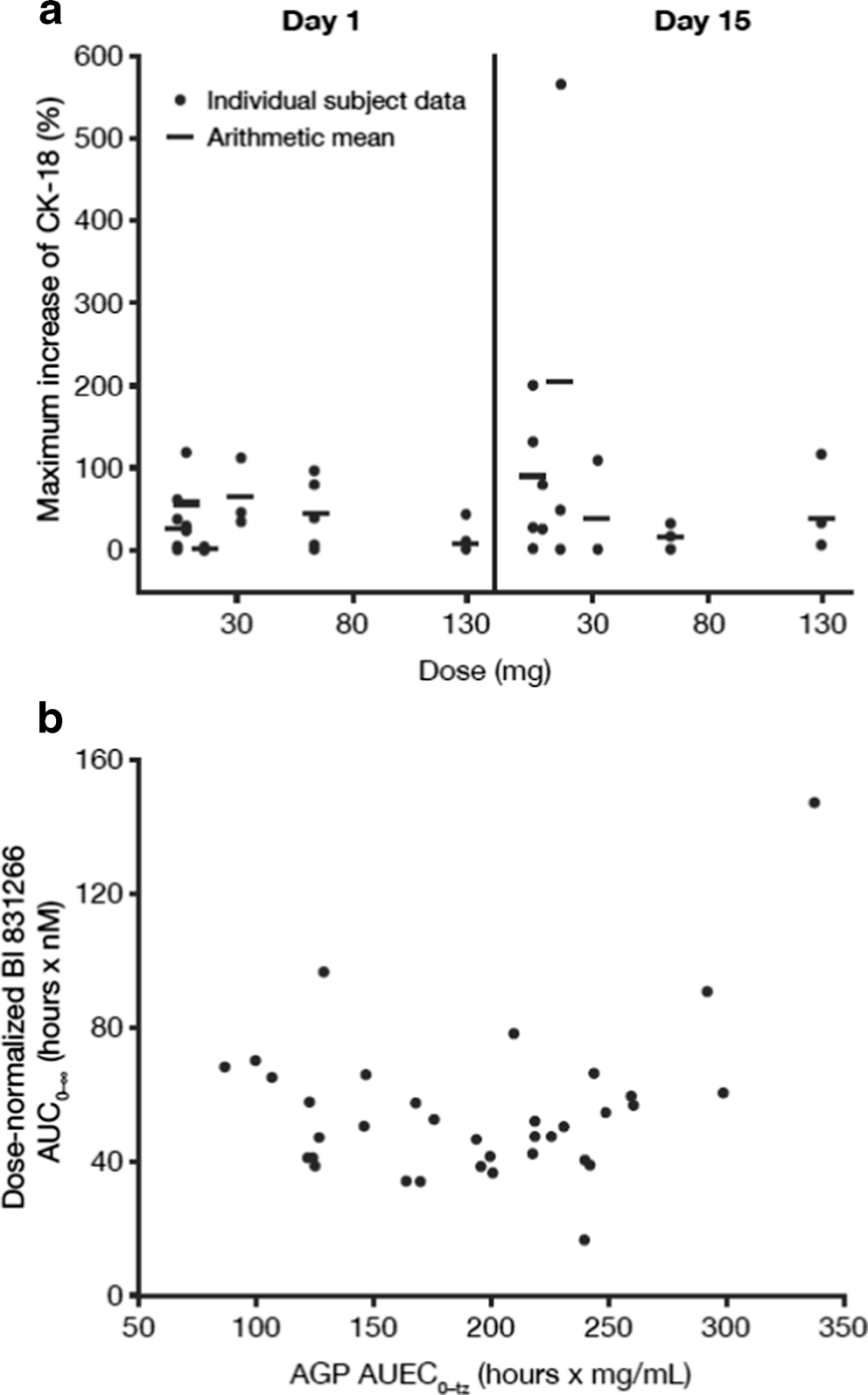
**a** Individual and mean maximum percentage increase in caspase-cleaved CK-18 plasma concentrations from baseline after the first 2 infusions of BI 831266. **b** Individual dose-normalized BI 831266 AUC_0-∞_ versus AGP AUEC_0-tz_, excluding abnormal PK profiles. *CK*-*18* cytokeratin-18, *AGP* alpha-1-acid glycoprotein, *AUC*_0-∞_ area under the plasma concentration–time curve over the time interval from 0 extrapolated to infinity, *AUEC*_*0*-*tz*_ area under the effect curve to the last evaluable timepoint, *PK* pharmacokinetic

Plasma concentrations of caspase-cleaved CK-18 showed variability and no consistent association with the dose of BI 831266 (Fig. 3a).

It has been reported that the exposures of some compounds are related to the concentration of AGP in patients [16, 17]. Therefore, BI 831266 dose-normalized AUC_0-∞_ exposure was plotted against the overall AGP exposure assessed as the area under the effect curve to the last evaluable time point (AUEC_0-tz_)_,_ excluding AUC_0-∞_ values from patients with abnormal PK profiles (Fig. 3b). Although the patient with the highest AGP concentrations also had the highest BI 831266 AUC_0-∞_, the overall data showed no trend of BI 831266 exposure with AGP concentration.

### Antitumor activity

One patient (4.0 %) with cervical cancer, who was treated with 32 mg BI 831266, experienced a confirmed PR according to RECIST, with a duration of 141 days and PFS of 414 days. The patient initially experienced stable disease (SD) during the first 9 cycles, and was first documented to have a PR after cycle 10. The patient completed a total of 14 cycles and remained in PR but stopped treatment due to serious AEs of gangrene and multi-organ failure.

**Fig. 4:**
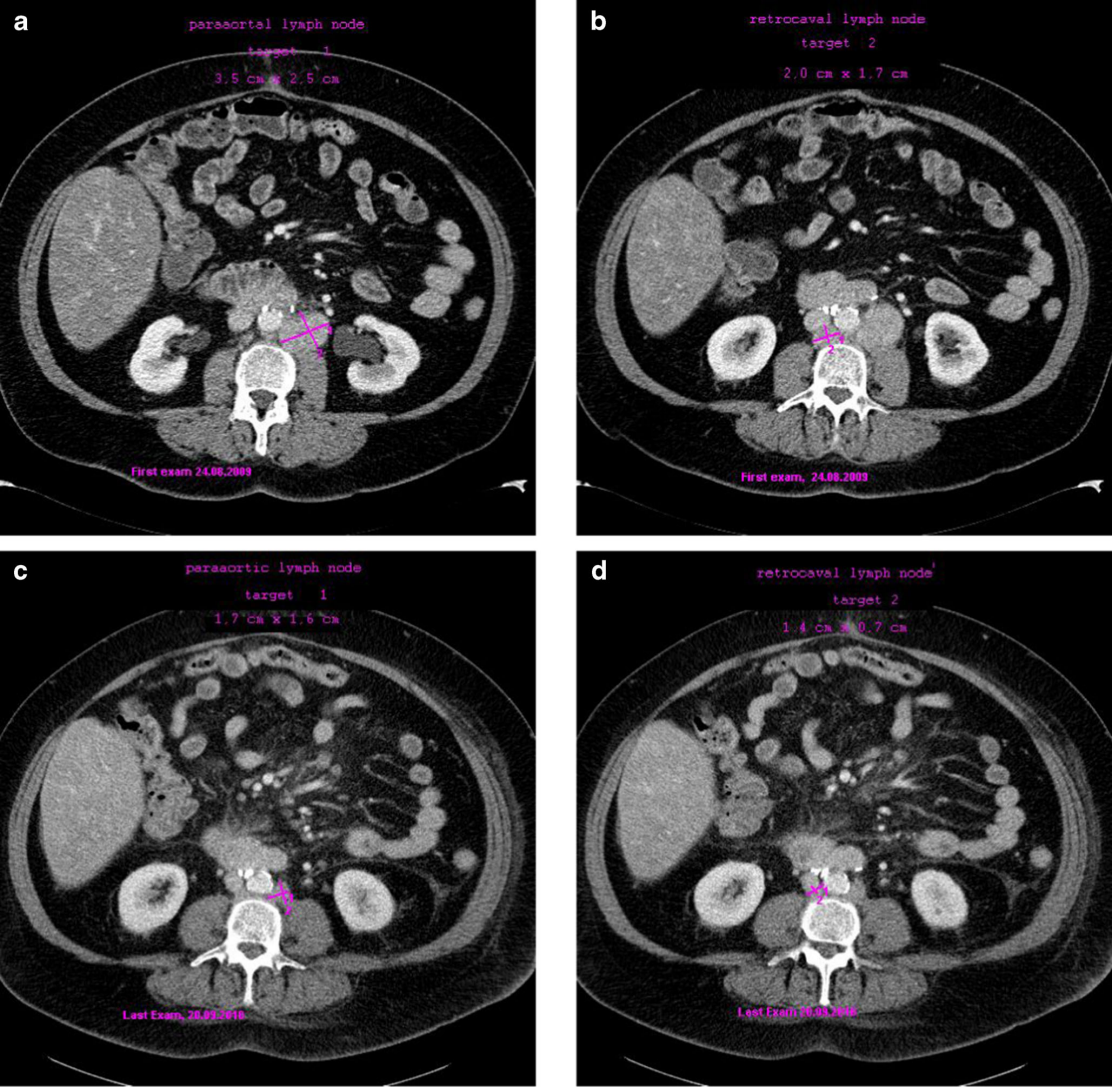
CT scans of a patient with cervical cancer who experienced a confirmed PR. **a** and **b**: baseline scans; **c** and **d**: following 14 cycles of treatment with BI 831266. PR was first documented after cycle 10. *PR* partial response

CT scans showing tumor shrinkage in this patient are shown in Fig. 4. An additional 4 patients (16.0 %; colorectal cancer: *n* = 2; pancreatic cancer: *n* = 1; bladder cancer: *n* = 1) experienced SD according to RECIST as best response. PFS among these patients ranged from 78 to 274 days. PFS in the 20 patients (80.0 %) without an objective RECIST response or SD ranged from 21 to 94 days.

The serious AEs observed in this one patient with PR were not considered to be treatment-related. The patient had suffered from hypercoagulability even before entering the clinical trial with BI 831266. The patient had had pulmonary embolization more than 1 month before the first drug application with BI 831266. After initial heparinization, the patient received oral anticoagulation with phenprocoumon. Nevertheless, the patient developed a massive thrombosis of the entire left lower extremity that extended to the pelvis, representing a 3-level thrombosis and also a partial thrombosis of the right posterior tibial vein. Despite recommencement of low molecular weight heparinization, the patient developed a gangrene of the left lower extremity, presenting as phlegmasia coerulea dolens, which necessitated the amputation of the left lower extremity that resulted in death due to multi-organ failure.

## Discussion

This study was discontinued prematurely primarily for two reasons: firstly, a lack of objective responses demonstrated by a sister front-runner compound developed by Boehringer Ingelheim (BI 811283), another intravenously-administered Aurora kinase B inhibitor [12, 18]. In a phase 1 study of BI 811283 involving a study population similar to the present study (121 patients with a variety of solid malignancies), no objective responses were seen and SD was only achieved in a third of the patients. Secondly, a new oral Aurora kinase inhibitor was being developed that would offer significantly improved convenience for patients [19] compared with the mode of administration of BI 831266 and BI 811283, which had to be administered via a 24-h continuous infusion through a central line.

Although reaching clinical activity in the form of responses does not typically represent a primary objective of a phase 1 trial, the fact that only 1 out of 25 patients experienced an objective response of the level of a PR let us examine the entire test assumption. In order to find out whether the anticipated mode of action of the drug and member of that class of drugs, respectively, was accurate, the measurement of histone H3 phosphorylated on serine 10, a well characterized substrate of Aurora kinase B, was performed. Pharmacodynamic analyses of skin biopsy samples demonstrated a reduction of pHH3 following treatment in some patients, suggesting that there was evidence of Aurora kinase inhibition. Analysis of the epidermal expression of the mitosis marker pHH3 as an indicator of Aurora kinase B inhibition assumes that BI 831266, at a dose of 130 mg, is biologically active, with an antimitotic mechanism of action. A consistent and robust reduction of approximately 70 % from baseline was observed in the number of epidermal cells expressing pHH3 from skin biopsies in all 6 evaluable patients treated at 130 mg BI 831266. However, a dose relationship was not clear; this is partly due to interpatient variability in pHH3 expression in some dose cohorts which precludes a conclusive observation. The decrease in the number of pHH3+ cells observed at the highest dose level of BI 831266 was consistent with selective Aurora kinase B inhibitory activity, which has been observed previously with other Aurora kinase B inhibitors in preclinical studies (data on file, Boehringer Ingelheim) [20–24]. We have therefore demonstrated the mechanism of action of BI 831266 in this trial. However, due to the small number of responders in this study, an exploratory analysis to determine a correlation between pHH3 and clinical outcome was not possible. Therefore, proof of principle in terms of sufficient Aurora kinase B inhibition leading to corresponding tumor shrinkage could not be proved in this trial.

A next step in the cascade of necessary proofs is the one of the mode of action-based consequent proof of a biologic effect downstream of the triggered initial drug target. As such, Aurora kinase B-induced apoptosis was investigated. Plasma concentrations of caspase-cleaved CK-18, a marker of apoptosis [25, 26], were highly variable and did not appear to be related to exposure to BI 831266. This result does not indicate that BI 831266 is substantially active. In this context, it has to be admitted that the molecular pathways through which tumor cells undergo cell death in response to mitotic arrest are not well defined. Whereas apoptosis, which is observed with many antimitotic agents, is a process well characterized by the activation of caspases, an alternative mechanism of cell death, termed mitotic catastrophe, is much less elucidated [27].

In order to exclude the possibility that this lack of activity was, at least in part, also the efflux of variability or insufficiency of exposure of tumor cells to the BI 831266, detailed PK investigations were undertaken. Plasma PK profiles showed extremely high variability, reflected in exposure parameters during the infusion, with some patients having much higher maximum measured concentration (C_max_) and AUC values than others. Therefore, dose proportionality could not be determined. Ten concentration-time PK profiles, rising and declining extremely rapidly, were incompatible with what was to be expected. This type of profile is not typically observed, even in drug-drug interaction trials. Drug-drug interactions may change the magnitude of a PK profile, but they usually do not change the basic shape of a profile in this manner. The reason for these profiles could not be established from the available data. Without these profiles, dose-normalized AUC_0-∞_ and C_max_ values appeared independent of dose (data not shown), suggesting that the increase in exposure was near linear at least, when the 64 mg dose level was excluded. However, the high variability and exclusion of the PK profiles preclude any definitive conclusions being drawn, especially with regard to correlation between PK and efficacy. Preclinical efficacy studies using various tumor models have shown that a target steady-state concentration of BI 831266 required to reach full antitumor activity was 80–153 nM, depending on the tumor model (data on file, Boehringer Ingelheim). In this phase 1 study, the geometric mean C_max_ at steady state of the 16 mg dose cohort was 280 nmol/L, which is already above the target exposure based on preclinical models. Due to high variability in PK parameter values, but nonetheless also due to the restricted clinical activity, conclusions about PK correlation with efficacy could not be established.

In contrast thereto, an assessment of the safety data from the present trial revealed a dose effect for BI 831266. Patients who received the highest dose tested in this trial (130 mg BI 831266) experienced treatment-related hematologic AEs, which were not observed in patients treated at lower dose levels. Treatment-related alopecia was also reported more frequently in the 130 mg BI 831266 cohort than at lower doses.

One PR was reported in this study, with SD seen in 16 % of patients. This is consistent with data from the phase 1 studies of other Aurora kinase inhibitors, most of which are inhibitors of Aurora kinase A, B, or all 3 mitotic kinases; SD was the best response in the majority of these studies [28–35]. Only 2 Aurora kinase inhibitors have reported a PR in their phase 1 studies [28, 30]; 1 of these was with the use of granulocyte colony-stimulating factor which allowed a higher dose of the compound to be administered [30]. Hematologic toxicity, an expected class effect, was a common DLT in all of these studies, preventing further dose escalation. To date, the most prominent efficacy of an Aurora kinase inhibitor has been observed in hematologic malignancies, specifically in acute myeloid leukemia where a superior objective clinical response rate was seen with barasertib treatment compared with low-dose cytosine arabinoside treatment in the control arm [36].

This leads to the fundamental open question of whether overall this class of compounds is sufficiently attractive and different from other already existing drugs to be further developed and in case of affirmation, which of the representatives should be selected and how they should be further developed.

Antimitotic therapies such as vinca alkaloids, taxanes, or even epothilones directed against tubulin and its homeostasis are known as highly effective compounds. Various substances mainly interfering with distinct functions in the mitotic process belong to different druggable target classes such as mitotic kinesins, polo-like kinases, and Aurora kinases [27]. No patient selection based on the molecular characterization has been accomplished to-date. This may be on one hand due to the assumption that increased mitosis is an ubiquitous phenomenon in all cancer populations, although not substantiated by data [37], and on the other hand that we do not know yet which tumors are likely to respond to a particular targeted inhibitor of mitosis due to our incomplete understanding of the respective downstream mechanisms. So, for example, it is unclear whether response to Aurora kinase inhibition depends on the p53 status of these tumors [38].

As with most therapeutic scenarios in oncology, particularly in solid tumors, the combination of drugs has proven to be more effective than monotherapy. Since Aurora kinase B specifically regulates the spindle checkpoint, combination with substances that target the mitotic spindle and that depend on the spindle checkpoint for activity (such as taxanes) could have been interesting to evaluate [39]. The combination with substances that require exposure during other phases of the cell cycle may represent an advantage, since distinct from traditional antimitotic agents Aurora B inhibitors do not arrest cells in mitosis, but let them continue to cycle. Combining Aurora B inhibition with Aurora A inhibition could therefore be considered in principle. However, an example of lack of efficacy of simultaneously targeting both enzymes has been demonstrated on pancreatic cancer cells [40]. Increased inhibition of Aurora kinase B activity with barasertib in combination with irinotecan and gemcitabine in a sequence-dependent manner (with barasertib before chemotherapy) and increased induction of apoptosis has been demonstrated in colorectal and pancreatic cell lines [41, 42]. It is a matter of speculation whether potentiation may be possible at doses below those inducing limiting neutropenia.

The very limited clinical activity observed in our trial compares well to the overall modest clinical activity of other Aurora kinase B inhibitors. This limitation is not even restricted to the entire family of Aurora kinase inhibitors but can be identified as a general feature of all inhibitors targeting the process of mitosis, including kinesins, and polo-like kinases [43–45]. This leads to the fundamental question of whether the basic theorem that tumors are characterized by short doubling times and high proliferation rate is correct. Although a plethora of preclinical data represent the basis of that belief, insight in patient data in comparison reveal the opposite [37]. Whereas the means and medians of tumor doubling times for five of the most prevalent cancer entities are accumulating at about 5 days, the results for patient data vary from about 100 to 400 days. The fact that obviously only a very small sub-fraction (<1 %) of solid tumors are undergoing mitosis seems to be a rather simple explanation of why compounds interfering with the mitotic process are generally so ineffective.
